# Should We Reconsider the Necessity of a Refinement of Prostate Cancer Risk Classification and Radiotherapy Treatment Strategy? Experiences from a Retrospective Analysis of Data from a Single Institution

**DOI:** 10.3390/jcm10010110

**Published:** 2020-12-30

**Authors:** Viktória Temesfői, Róbert Herczeg, Zoltán Lőcsei, Klára Sebestyén, Zsolt Sebestyén, László Mangel, Miklós Damásdi

**Affiliations:** 1 Lab-on-a-Chip Research Group, János Szentágothai Research Center, University of Pécs, H-7624 Pécs, Ifjúság útja 20, Hungary; temesfoi.viktoria@pte.hu; 2 Department of Laboratory Medicine, Medical School, University of Pécs, H-7624 Pécs, Ifjúság útja 13, Hungary; 3 Bioinformatics Research Group, Genomic and Bioinformatics Core Facility, János Szentágothai Research Center, University of Pécs, H-7624 Pécs, Ifjúság útja 20, Hungary; herczeg.robert@pte.hu; 4 Department of Oncotherapy, Clinical Centre, Medical School, University of Pécs, H-7624 Pécs, Édesanyák útja 10, Hungary; locsei.zoltan@pte.hu (Z.L.); klara.sebestyen@kk.pte.hu (K.S.); sebestyen.zsolt@pte.hu (Z.S.); 5 Urology Clinic, Clinical Centre, Medical School, University of Pécs, H-7621 Pécs, Munkácsy Mihály utca 2, Hungary; damasdi.miklos@pte.hu

**Keywords:** prostate cancer, radiotherapy, androgen deprivation therapy, therapy optimization

## Abstract

Background: Radiation therapy has undergone significant technical development in the past decade. However, the complex therapy of intermediate-risk patients with organ-confined prostate carcinoma still poses many questions. Our retrospective study investigated the impact of selected components of the treatment process including radiotherapy, hormone deprivation, risk classification, and patients’ response to therapy. Methods: The impact of delivered dose, planning accuracy, duration of hormone deprivation, risk classification, and the time to reach prostate-specific antigen (PSA) nadir state were analyzed among ninety-nine individuals afflicted with organ-confined disease. Progression was defined as a radiological or biochemical relapse within five years from radiotherapy treatment. Results: We found that 58.3% of the progressive population consisted of intermediate-risk patients. The progression rate in the intermediate group was higher (21.9%) than in the high-risk population (12.1%). Dividing the intermediate group, according to the International Society of Urological Pathology (ISUP) recommendations, resulted in the non-favorable subgroup having the highest rate of progression (33.3%) and depicting the lowest percentage of progression-free survival (66.7%). Conclusion: Extended pelvic irradiation on the regional lymph nodes may be necessary for the ISUP Grade 3 subgroup, similarly to the high-risk treatment. Therapy optimization regarding the intermediate-risk population based on the ISUP subgrouping suggestions is highly recommended in the treatment of organ-confined prostate cancer.

## 1. Introduction

Due to early detection and a widely adopted screening policy, prostate malignancies are generally recognized in the early stages. Options for treatment for curative intent of localized prostate cancer (PC) include active surveillance, radical prostatectomy, curative external beam radiation therapy (EBRT), and brachytherapy [1]. Androgen deprivation therapy (ADT) combined with radiation have been shown to increase survival in males with intermediate and high risk of relapse [2,3], as well as local and systemic effects. Whether the increased efficacy of combining ADT and EBRT is the result of an improved local treatment (radiosensitizing effect) or the systemic eradication of micro-metastases, or the combination of both, remains as of yet, an unanswered question. The administration of ADT leads to a decrease in volume of the entire prostate gland, which is seemingly advantageous considering radiotherapy target volume determination. Additionally, an increasing number of patients with favorable and unfavorable intermediate- and high-risk adenocarcinoma of the prostate have been treated using a combination of ADT and primary EBRT [4,5], which is likely an appropriate and effective form of treatment.

Over the past two decades, advances in radiotherapy technology have been of great importance in the conventional forms of treatment combating various cancers with different radiosensitivities. In consideration of the dose escalation methods, the use of image-guided, intensity-modulated techniques generally reduces the potential side effects to an acceptable level. As a result of these technological advances, radiotherapy has undergone a major transformation over the past decade. Recent results have also shown that new techniques and dose escalation have increased the potential of disease-free survival [6,7].

The Oncotherapy Center, University of Pécs has nearly ten years of experience and clinical results regarding advanced radiotherapy for PC. Notably, a total of 267 localized prostate adenocarcinoma patients underwent EBRT, between 2012 and 2013, at the Institute of Oncotherapy, Clinical Center, University of Pécs, based on multidisciplinary decision and risk-adapted selection. The patients were treated featuring a new technological and therapeutic approach using a state-of-the-art radiotherapy device Novalis Tx (Varian, Palo Alto, CA, USA). From this cohort, 99 patients were included in our study in which we investigated the impact of selected components, including radiotherapy data, hormone deprivation, risk classification, and the patients’ response to therapy regarding progression-free survival.

## 2. Materials and Methods

### 2.1. Enrolled Patients

Ninety-nine individuals with an organ-confined disease were selected from a total of 267 treated PC patients from 1 January 2012 to 31 December 2013. The ninety-nine individuals were selected based on the fact that full documentation of their follow-up was available in the databases of the Clinical Center, University of Pécs. At the time, in several cases, the duration of neoadjuvant and adjuvant ADT did not consistently follow current recommendations, and it varied on a wider spectrum than following the introduction of more improved directives. Since 2011, standardization has increased throughout Hungary. Patients’ examinations and follow-up of the selected patients were performed at the Urology Clinic and also at the Department of Oncotherapy, University of Pécs. The work was implemented in accordance with the Helsinki Declaration and was approved by the Regional Ethics Committee of the Medical School, University of Pécs, ethical license code IV/7266-1/2020/EKU.

### 2.2. Determination of Treatments

The risk-adapted treatment was defined based on the 2009 guidelines and recommendations of the European Association of Urology (EAU) regarding the duration of hormone deprivation therapy and were designed to target low-, intermediate- and high-risk patients [1,8]. The most frequently-administered drugs to target and block androgen production at the time of treatments were orally-administrated anti-androgens (bicalutamide and nilutamide) and parenterally-administrated luteinizing hormone-releasing hormone (LHRH) analogs (leuprorelin and goserelin). The planning and implementation of patient radiotherapy was based on the guidelines of the European Society for Radiotherapy and Oncology (ESTRO) [9] in force at the time. According to the ESTRO guideline, the delivered dose to patients with low-risk PC was 74 gray (Gy) in total, in 2 Gy daily fractions to the prostate and to the base of the vesicula seminalis. Intermediate-risk patients received 78 Gy total dosage targeted to the prostate and to the vesicula seminalis. In the high-risk group, 78 Gy was delivered to the prostate and vesicula seminalis, however, these patients received extended radiation to the pelvic lymph node region elevating to 46 Gy. The dose limits regarding risk organs were defined based on the Quantitative Analyses of Normal Tissue Effects in the Clinic (QUANTEC) criteria [10], and the dose coverage of the target region was calculated using the International Commission on Radiation Units and Measurements (ICRU) 83 recommendations [11].

### 2.3. Definition of Progression

Patients who were diagnosed with radiological (changes in size and/or number of novel metastatic lesions) or biochemical relapse (exceeding the prostate-specific antigen (PSA) cut off level of 2.0 ng/mL) within five years following radiotherapy treatment were considered to be afflicted with a progressive disease. Progression as a dependent variable was converted into binary data; ”0” represented no progression and ”1” depicted progression.

### 2.4. Explanatory Variables

Covariates were selected to cover four main areas of the study, including radiotherapy parameters (delivered dose and accuracy of planning), hormone deprivation therapy (treatment duration), risk classification, and patient’s response to therapy (time required to reach a PSA nadir state). The delivered dose was represented in gray units (Gy), planning accuracy was based on the V95 value representing the accuracy of the design and treatment and depicted the percentage of the target volume (prostate or tumor) which received 95% of the delivered dose. The ADT duration included the months of neoadjuvant and adjuvant therapy. PSA nadir is defined as the lowest level of PSA measured following treatment.

### 2.5. Reclassification

The studied population was reclassified based on the suggestions of the International Society of Urological Pathology (ISUP) [12] and descriptive statistics were analyzed in the same manner as the prior original classification.

### 2.6. Machine Learning Models: Logistic Regression for Prediction

To effectively monitor the differences among the two type of risk classifications (original and ISUP recommended), we used the logistic regression-based supervised machine learning approach. In both cases, the dataset was split as follows: 75% of the data was defined as the training set and 25% served as the test set.

### 2.7. Statistical Analysis

Descriptive statistics were performed to summarize and characterize the available raw data. Due to a nonparametric distribution of each tested variable, median and interquartile ranges were included once a variable was continuous. Categorical data were interpreted as proportion and frequencies. In order to interpret the relationship of the variables, principal component analysis (PCA) was applied. Binary logistic regression (backward likelihood ratio, *p* < 0.1) was performed to reveal the influence of the covariates on the progression. A Cox proportional hazards regression analysis was used to investigate the association between the classification and the elapsed time to biochemical or radiological relapse in the risk groups. Statistical analyses were carried out using the SPSS v23 (IBM, Armonk, NY, USA) and machine learning models were built in R (R Foundation for Statistical Computing, Vienna, Austria). Prism software version 8 (GraphPad, San Diego, CA, USA) was used to visualize data.

## 3. Results

### 3.1. Intermediate-Risk Population Showed the Highest Percentage of Progression among the Categories

Interestingly, while investigating the disease progression in connection with risk classification, we found that the intermediate-risk group was represented in the progressive population with the highest proportion of the three categories (58.30%) and the highest progression rate was also detected within this population (21.90%) (Table 1).

**Figure 1:**
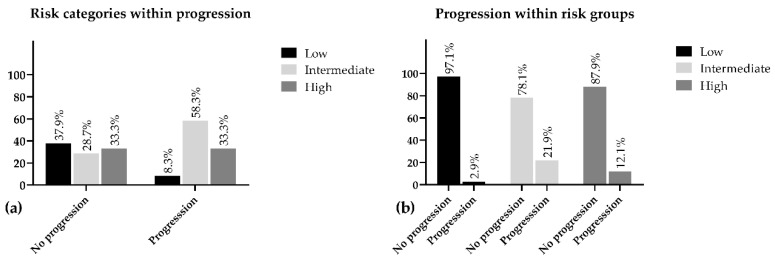
Percentage distribution of risk groups within progression (**a**) and progression within risk (**b**).

The low-risk patients depicted the lowest percentage of progression within group (2.9%) and represented 8.3% of the overall progression. Additionally, 97.1% of this population was progression-free during the five years of follow-up. Despite our expectations, we found the progression in the intermediate group was 9.8% higher as compared with the high-risk population. The intermediate group appeared to be the least represented (28.7%) in the progression-free population and only 78.1% of these patients did not face any advancements regarding PC, while this ratio in the high-risk group was 87.9% (Figure 1).

### 3.2. Relationship of the Variables

**Figure 2:**
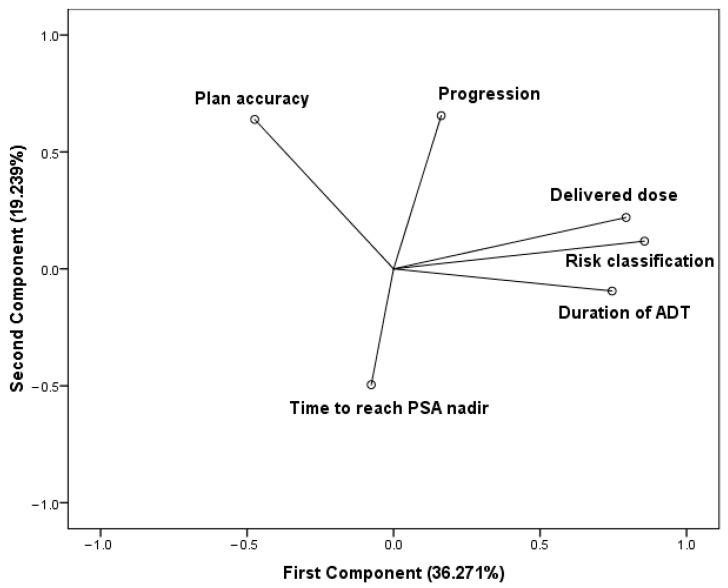
Components in rotated space. Principal component analysis (PCA) using varimax rotation method with Kaiser normalization. Rotation converged in three iterations.

The PCA was based on a correlation matrix of the variables. The number of principal components were selected by an eigenvalue (≥1). The first two principal components contribute 55.51% of the total variation in the dataset. The first component has strong positive associations with the delivered dose, the duration of the hormone deprivation treatment, and the risk classification, therefore, this component measures the quality of treatments. Since the delivered dose and ADT duration are determined based on the risk classification, these variables vary together. The accuracy regarding radiotherapy planning is in positive association with the second component. The elapsed time to reach the PSA nadir state seems to be in negative association with both components. Progression is positioned between the treatment quality cluster and plan accuracy and features a strong positive correlation with the second component (Figure 2).

### 3.3. Risk Classification Is the Most Associated with Progression among the Investigated Variables

We performed binary logistic regression in order to assess the effects of delivered dose during radiotherapy treatment, planning accuracy of radiotherapy, the duration of hormone deprivation treatment, the time during which the patient reaches the nadir value of PSA level, and the classification into the risk groups on the progression of the neoplastic prostatic disease. The definition of progression is defined in the Methods section. The properties in reference to the explanatory variables are listed in Supplementary Table S1. Our methodology correctly classifies the outcome in 88.8% of the cases.

#### Backward Stepwise Logistic Model

The included variables in the first model of stepwise statistics are the following: (constant), delivered dose, plan accuracy, risk (categories 1, 2, and 3), ADT duration, time required to reach the PSA nadir state. The fifth model only includes the risk classification, which suggests, in reference to the parameters involved, that risk classification may be most associated with progression in our testing circumstances (Table 2). However, it should be mentioned that the fifth model explains 12.3% of the variance in progression (Supplementary Table S2).

### 3.4. Intermediate-Risk Group Tends to Form Two Subgroups When Risk Classification Is Reconsidered According to the International Society of Urological Pathology (ISUP) Recommendations

The intermediate-risk group is divided into two (favorable and non-favorable) subgroups based on the Gleason score, which are represented as Intermediate 1 and 2 (Table 3). The properties of explanatory variables following reclassification are listed in Supplementary Table S3.

**Figure 3:**
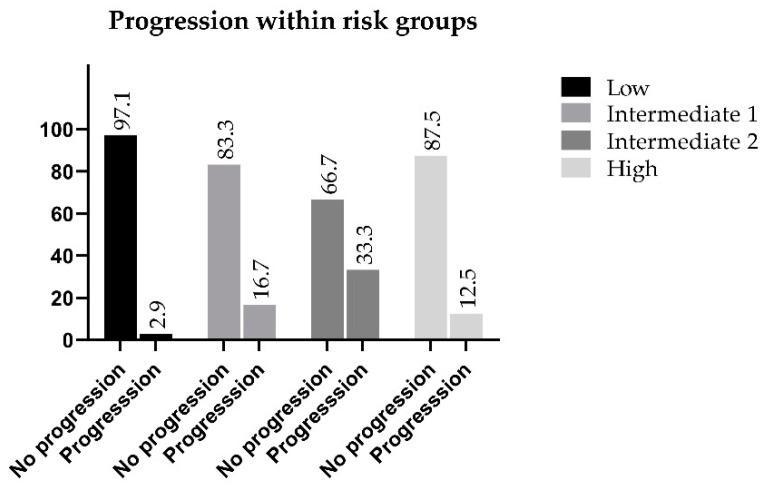
Progression within the reclassified risk groups.

The highest percentage of progression is observed within the Intermediate 2 risk group, which is considered to be ISUP Grade 3. Thirty-three percent of these patients developed a progressive disease within five years from treatment, while in the favorable intermediate group, the progression rate was only 16.7% as compared with 12.5% of the high-risk population that showed progression, which was 4.2% lower than the favorable intermediate group, and 20.8% lower than the non-favorable category. Progression-free survival of the Intermediate 2 patients was the lowest among the categories (66.7%) (Figure 3).

### 3.5. The Progression-Free Survival Probability of the Intermediate Risk Patients (ISUP Grade 2 and 3) Is Worse than the High Risk Group

**Figure 4:**
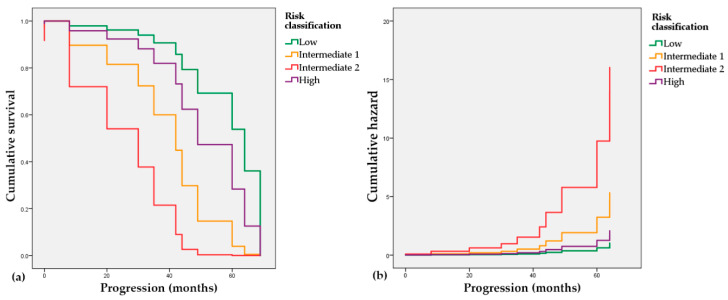
Cox proportional hazards regression. (**a**) Survival probability of patients in the reclassified risk groups; (**b**) Cumulative hazard of progression in the reclassified risk groups.

In order to assess the connection of the risk classification and the elapsed time to the progression event (radiological or biochemical), we used the Cox proportional hazards model. The low-risk group with a negative coefficient (B = −0.711, *p* = 0.541) is associated with a decreased hazard and longer progression-free times, while the Intermediate 1 and 2 groups with positive coefficients (B = 0.942, *p* = 0.309 and B = 2.044, *p* = 0.059, respectively) have an association with increased hazard and shorter predicted time to progression. In Figure 4, it is also visible that both intermediate groups depict worse trends regarding progression-free time intervals than the high-risk group.

### 3.6. Data Validation by Machine Learning Approach

**Figure 5:**
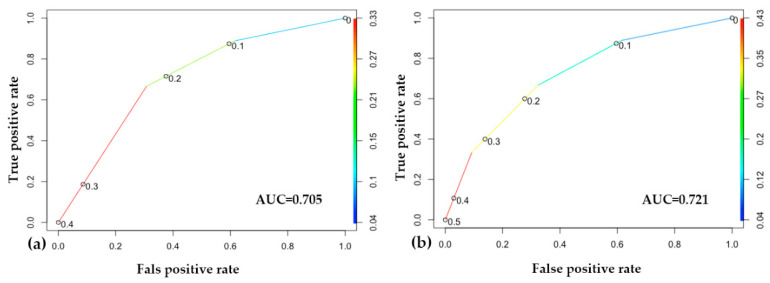
Results of the machine learning approach. (**a**) Receiver operating characteristic (ROC) curve of the original classification-based model; (**b**) ROC curve of the model based on the International Society of Urological Pathology (ISUP) reclassification. In both of the models, at the point (0.6, 0.9), we correctly label about 90% of the cases, with a 60% false positive rate. In the middle, around (0.3, 0.8), we correctly label about 80% of the cases, with a 30% false positive rate.

The performance of the two machine learning models built on the two different risk classifications is very similar. According to the accuracy score, both of our models correctly classify 72% of the outputs. Calculating the area under the receiver operating characteristic curve (ROC AUC), the predictive accuracy of the second model, which was built on the ISUP recommended system, is slightly higher (AUC 0.721) than that of the first model, which was based on the original classification (AUC 0.705), which implies that the separation of the intermediate-risk group into favorable and non-favorable subgroups provides a basis in which the classification performance of the machine learning model is higher and the outcome (progression) can be predicted more accurately (Figure 5).

## 4. Discussion

According our calculations, 12.3% of the variance in progression is explained by the risk classification. This result raises the necessity to acknowledge other factors which are not represented in our study, such as individual biological variance or the general social and health status regarding patients. Classification of the analyzed individuals, in 2012, was guided by the pathological report, clinical status, laboratory parameters, computed tomography (CT) and scintigraphy investigations. The histopathological examinations were performed in a single institute, and during the processing of the needle biopsy, the involvement of the core samples, the Gleason Grade, and the malignant tissue type (adenocarcinoma) were determined. No further subtypes of adenocarcinoma could be identified. Cribriform architecture was not investigated in the histopathological findings, and thus it could not be considered to be a prognostic factor in the case of Gleason Grade 4. The ISUP classification was set up based on the knowledge of the PSA level, Gleason Grade, and local status. The introduction of the multiparametric MR imaging resulted in a substantial improvement regarding personal diagnostics, in particular, the definition of the primary tumor status and in the detection of lymph node metastases [13] In this study, we placed special emphasis on possible improvements of risk classification-based treatment selection.

According to the current understanding and regulations, radiotherapy is administered to patients as a curative treatment of PC, while ADT is an additional form of treatment. In the field of radiotherapy, EBRT including intensity-modulated radiation therapy (IMRT) and volumetric-modulated arc therapy (VMRT) play a key role in the treatment of organ-localized or locally advanced PC [14].

The addition of ADT to radiotherapy improves biochemical and survival outcome in patients with intermediate- and high-risk or locally advanced disease, yet not in the low-risk group [15]. Jones et al. (Radiation Therapy Oncology Group, RTOG 94-08) studied the impact of short-term ADT in localized PC patients. Among individuals with ISUP Grade 2–3 PC, the use of short-term neoadjuvant ADT for four months prior to and during radiotherapy was associated with significantly decreased disease-specific mortality and increased overall survival. The benefit was primarily seen in the intermediate-risk group, yet not in the low-risk group [16]. Another interesting question is the duration of ADT in combination with radiotherapy. The 2020 EAU guideline offers active surveillance, radiotherapy alone, or nerve-sparing prostatectomy alone to patients with a life expectancy over ten years and low-risk disease. The EAU guideline also recommends radiotherapy, plus four to six months ADT to intermediate-risk patients, while to the high-risk patients, radiotherapy plus two to three years ADT is recommended. IMRT combined with short-term ADT (four to six months) can be given to intermediate-risk subjects suitable for ADT, however, for cases unsuitable for ADT, the alternative treatment is IMRT or VMAT at an escalated dosage rate (76–80 Gy) or a combination of IMRT or VMAT and brachytherapy. For high-risk patients with localized PC, IMRT or VMAT at an escalated dosage rate plus long-term ADT is the preferable combination [17].

The duration of ADT in the high-risk group was analyzed in different clinical studies. The short-term ADT administration combined with radiotherapy did not improve overall survival in high-risk localized PC. The long-term ADT (at least two to three years) is currently recommended for these individuals [18,19].

According to a large clinical trial, the primary ADT for low-risk PC patients did not improve overall survival [15]. However, D’Amico et al. reported results of a large retrospective study in reference to males treated with three-dimensional conformal radiation therapy (3D-CRT) with or without ADT for low-, intermediate-, and high-risk PC. The five-year PSA relapse-free survival of low-risk PC was 92% with the addition of ADT versus the ratio of 84% without ADT. Interestingly, these results demonstrated potential benefits of the short-term hormone withdrawal therapy in the low-risk group as well [20]. The optimal timing for androgen deprivation has not been precisely determined. The combination of radiotherapy with LHRH-ADT has definitively proven its superiority as compared with radiotherapy alone followed by deferred ADT on relapse in intermediate- and high-risk PC group, as shown by phase III randomized clinical trials [21]. Currently, approximately two to three months of neoadjuvant therapy is likely to be the optimal strategy in an intermediate-risk PC group followed by eight to ten months of adjuvant treatment. However, regarding favorable intermediate-risk patients, the latest National Comprehensive Cancer Network (NCCN) guideline recommends radiotherapy alone [5].

Studies have shown a survival benefit regarding patients with advanced disease when longer adjuvant ADT was applied (three years). In a 6.4 year follow-up study, the overall mortality was higher with short-term ADT (six months) than with long-term treatment (thirty-six months). The authors stated that long-term ADT combined with radiotherapy should be the gold standard for high-risk PC patients [22].

The common side effects associated with ADT such as skeletal, metabolic and cardiovascular complications, sexual dysfunction, and mood disorders clearly highlight the need for an optimal schedule for ADT in combination with EBRT. To achieve this, a treatment schedule may have to be highly individualized based on patient-specific potential vulnerability to adverse events [23].

In our practice, we initiated the administration of an LHRH analog following two weeks of anti-androgen (50 mg bicalutamide) pretreatment as a complete androgen blockade neoadjuvant ADT prior to radiotherapy. Following one month of anti-androgen treatment, long-term LHRH analog administration was continued based on the risk-adapted categories.

Patients in the intermediate-risk group are considered to be a clinically heterogeneous population. Subgroup definitions possibly provide a better framework for their treatment. The current ISUP classification system divides patients with a Gleason score of seven into two subgroups, i.e., ISUP 2 with primary Gleason 3 and ISUP 3 with primary Gleason 4. This results in low/favorable (ISUP Grade 2) and high/unfavorable (ISUP Grade 3) intermediate-risk subgroups, which suitably reflect the heterogeneity that is representative of this population and the differences regarding the behavior of the tumor [17].

In analyzing the intermediate-risk group, we experienced worse results in progression-free survival as compared with high-risk cases. The prognostic classification fundamentally determines the treatment, both the duration of neoadjuvant and adjuvant ADT, and the need for irradiation. The high-risk group of patients received high-dosage radiotherapy, including the regional pelvic lymph nodes. In contrast, patients in the intermediate-risk group, even with non-favorable neoplastic processes, did not receive irradiation to the pelvic nodes. In the directives and guidelines that were in force in 2012, it was not obviously stated whether these individuals should be given pelvic irradiation. While examining in greater detail the three cases that were reclassified into the non-favorable intermediate category in our study, we found all the individuals developed bone metastases. This data suggests that metastatic processes most likely affected the pelvic lymph nodes prior to the formation of distant tumors, as this region is a potential first step for cancer cells to spread throughout the lymphatic system. A risk of nodal metastases over 5% (based on the Briganti nomogram) is an indication to perform extended regional nodal dissection [24]. The probability of having positive lymph nodes in intermediate-risk PC is between 3.7 and 20.1%. Extended lymphadenectomy should be performed in this group when the estimated risk for pN+ exceeds 5%, according to the Briganti nomogram [25]. This data also emphasizes the importance of the supply of pelvic lymph nodes in some intermediate-risk cases.

It is necessary to mention that both classification and treatment selection are influenced by the availability of imaging opportunities (PET, CT, and MR). At the time, the most questionable portion of the diagnosis was the determination of the size and extent of the primary tumor and the detection of pelvic lymph node involvement, which fundamentally influenced the separation of intermediate- and high-risk PC and the definition of subgroups which could be formed within the intermediate-risk population.

## 5. Conclusions

The results of this study call attention to the fact that it has been repeatedly stated in oncology treatment that personalized therapy cannot be performed without precise, accurate evaluation of the histopathological and imaging examination obtained from malignant processes [26]. Patients in the intermediate-risk group can be considered to be a highly heterogeneous population, therefore it is recommended to separate the low and high ISUP stages already defined in the new urologic oncology guidelines, to adjust the neoadjuvant and adjuvant ADT and to extend the field similarly to the high-risk group dose delivery during radiotherapy.

## Figures and Tables

**Table 1:** Cross-tabulation of risk classification and disease progression. Appearance of risk categories in progressive and non-progressive groups and percentage distribution of progression within risk groups.

		Risk			
		Low	Intermediate	High	Total Cases
No progression	Number of cases	33	25	29	87
	% Within progression	37.90%	28.70%	33.30%	100%
	% Within risk	97.10%	78.10%	87.90%	87.90%
	% Of total	33.30%	25.30%	29.30%	87.90%
Progression	Number of cases	1	7	4	12
	% Within progression	8.30%	58.30%	33.30%	100.00%
	% Within risk	2.90%	21.90%	12.10%	12.10%
	% Of total	1.00%	7.10%	4.00%	12.10%
Total cases	Number of cases	34	32	33	99
	% Within progression	34.30%	32.30%	33.30%	100.00%
	% Within risk	100.00%	100.00%	100.00%	100.00%
	% Of total	34.30%	32.30%	33.30%	100.00%

**Table 2:** Stepwise statistics of binary logistic regression (backward stepwise/likelihood ratio).

		Coefficient (B)	S.E.	Sig.
Step 1	Delivered dose	0.079	0.208	0.706
	Plan accuracy	0.289	0.304	0.343
	Risk 1	−0.910	1.495	0.543
	Risk 2	1.138	0.892	0.202
	Risk 3			0.098
	ADT duration (months)	0.009	0.018	0.622
	Time to reach PSA nadir (months)	0.000	0.044	0.995
	Constant	−37.204	35.104	0.289
Step 2	Delivered dose	0.079	0.208	0.706
	Plan accuracy	0.289	0.304	0.343
	Risk 1	−0.910	1.494	0.543
	Risk 2	1.138	0.891	0.201
	Risk 3			0.097
	ADT duration (months)	0.009	0.018	0.620
	Constant	−37.194	35.073	0.289
Step 3	Plan accuracy	0.285	0.300	0.343
	Risk 1	−1.223	1.246	0.326
	Risk 2	0.992	0.792	0.210
	Risk 3			0.090
	ADT duration (months)	0.010	0.018	0.579
	Constant	−30.585	29.738	0.304
Step 4	Plan accuracy	0.233	0.283	0.410
	Risk 1	−1.409	1.200	0.240
	Risk 2	0.871	0.756	0.249
	Risk 3			0.091
	Constant	−25.113	27.799	0.366
Step 5	Risk 1	−1.228	1.182	0.299
	Risk 2	0.996	0.742	0.180
	Risk 3 *			0.086
	Constant	−2.269	0.606	0.000

* *p* < 0.1.

**Table 3:** Cross-tabulation of the reclassified risk groups versus progression.

		Risk				
		Low	Intermediate 1	Intermediate 2	High	Total Cases
No progression	Number of cases	33	20	6	28	87
	% Within progression	37.9%	23.0%	6.9%	32.2%	100.0%
	% Within risk	97.1%	83.3%	66.7%	87.5%	87.9%
	% Of total	33.3%	20.2%	6.1%	28.3%	87.9%
Progression	Number of cases	1	4	3	4	12
	% Within progression	8.3%	33.3%	25.0%	33.3%	100.0%
	% Within risk	2.9%	16.7%	33.3%	12.5%	12.1%
	% Of total	1.0%	4.0%	3.0%	4.0%	12.1%
Total cases	Number of cases	34	24	9	32	99
	% Within progression	34.3%	24.2%	9.1%	32.3%	100.0%
	% Within risk	100.0%	100.0%	100.0%	100.0%	100.0%
	% Of total	34.3%	24.2%	9.1%	32.3%	100.0%

## Data Availability

The data presented in this study are available on request from the corresponding author. The data are not publicly available due to restrictions eg privacy or ethical.

## Supplementary Materials

The following are available online at https://www.mdpi.com/2077-0383/10/1/110/s1, Table S1: Properties of explanatory variables in the three risk groups, respectively, Table S2: Model summary of binary logistic regression (backward stepwise/likelihood ratio), Table S3: Properties of explanatory variables in the four risk groups reclassified by the ISUP recommendations.

## References

[1] Mottet N., Bellmunt J., Bolla M., Briers E., Cumberbatch M.G., De Santis M., Fossati N., Gross T., Henry A.M., Joniau S. (2017). EAU-ESTRO-SIOG Guidelines on Prostate Cancer. Part 1: Screening, Diagnosis, and Local Treatment with Curative Intent. Eur. Urol..

[2] Sharifi N., Gulley J.L., Dahut W.L. (2005). Androgen deprivation therapy for prostate cancer. JAMA.

[3] Widmark A., Klepp O., Solberg A., Damber J.-E., Angelsen A., Fransson P., Lund J.-Å., Tasdemir I., Hoyer M., Wiklund F. (2009). Endocrine treatment, with or without radiotherapy, in locally advanced prostate cancer (SPCG-7/SFUO-3): An open randomised phase III trial. Lancet.

[4] Schmidt B., Eapen R.S., Cowan J.E., Broering J.M., Greene K.L., Carroll P.R., Cooperberg M.R. (2019). Practice patterns of primary EBRT with and without ADT in prostate cancer treatment. Prostate Cancer Prost. Dis..

[5] Amit U., Lawrence Y.R., Weiss I., Symon Z. (2019). Radiotherapy with or without androgen deprivation therapy in intermediate risk prostate cancer?. Radiat. Oncol..

[6] Rudat V., Nour A., Hammoud M., Alaradi A., Mohammed A. (2016). Image-guided intensity-modulated radiotherapy of prostate cancer: Analysis of interfractional errors and acute toxicity. Strahlenther. Onkol..

[7] Treece S.J., Mukesh M., Rimmer Y.L., Tudor S.J., Dean J.C., Benson R.J., Gregory D.L., Horan G., Jefferies S.J., Russell S.G. (2013). The value of image-guided intensity-modulated radiotherapy in challenging clinical settings. Br. J. Radiol..

[8] Cornford P., Bellmunt J., Bolla M., Briers E., De Santis M., Gross T., Henry A.M., Joniau S., Lam T.B., Mason M.D. (2017). EAU-ESTRO-SIOG Guidelines on Prostate Cancer. Part II: Treatment of Relapsing, Metastatic, and Castration-Resistant Prostate Cancer. Eur. Urol..

[9] European Society for Radiotherapy and Oncology (ESTRO) Guideline. https://www.estro.org/Science/Guidelines.

[10] Marks L.B., Yorke E.D., Jackson A., Ten Haken R.K., Constine L.S., Eisbruch A., Bentzen S.M., Nam J., Deasy J.O. (2010). Use of normal tissue complication probability models in the clinic. Int. J. Radiat. Oncol. Biol. Phys..

[11] International Commission on Radiation Units and Measurements Journal of the ICRU.

[12] Epstein J.I., Egevad L., Amin M.B., Delahunt B., Srigley J.R., Humphrey P.A., Committee G. (2016). The 2014 International Society of Urological Pathology (ISUP) Consensus Conference on Gleason Grading of Prostatic Carcinoma: Definition of Grading Patterns and Proposal for a New Grading System. Am. J. Surg. Pathol..

[13] Turkbey B., Mani H., Shah V., Rastinehad A.R., Bernardo M., Pohida T., Pang Y., Daar D., Benjamin C., McKinney Y.L. (2011). Multiparametric 3T prostate magnetic resonance imaging to detect cancer: Histopathological correlation using prostatectomy specimens processed in customized magnetic resonance imaging based molds. J. Urol..

[14] Quan E.M., Li X., Li Y., Wang X., Kudchadker R.J., Johnson J.L., Kuban D.A., Lee A.K., Zhang X. (2012). A comprehensive comparison of IMRT and VMAT plan quality for prostate cancer treatment. Int. J. Radiat. Oncol. Biol. Phys..

[15] Lu-Yao G.L., Albertsen P.C., Moore D.F., Shih W., Lin Y., DiPaola R.S., Yao S.L. (2008). Survival following primary androgen deprivation therapy among men with localized prostate cancer. JAMA.

[16] Jones C.U., Hunt D., McGowan D.G., Amin M.B., Chetner M.P., Bruner D.W., Leibenhaut M.H., Husain S.M., Rotman M., Souhami L. (2011). Radiotherapy and Short-Term Androgen Deprivation for Localized Prostate Cancer. N. Engl. J. Med..

[17] Mottet N., van den Bergh R.C.N., Briers E. (2019). EAU Guidelines edn. Presented at the EAU Annual Congress Barcelona. https://uroweb.org/guidelines/.

[18] Roach M., Bae K., Speight J., Wolkov H.B., Rubin P., Lee R.J., Lawton C., Valicenti R., Grignon D., Pilepich M.V. (2008). Short-term neoadjuvant androgen deprivation therapy and external-beam radiotherapy for locally advanced prostate cancer: Long-term results of RTOG 8610. J. Clin. Oncol..

[19] Denham J.W., Steigler A., Lamb D.S., Joseph D., Turner S., Matthews J., Atkinson C., North J., Christie D., Spry N.A. (2011). Short-term neoadjuvant androgen deprivation and radiotherapy for locally advanced prostate cancer: 10-year data from the TROG 96.01 randomised trial. Lancet Oncol..

[20] D’Amico A.V., Schultz D., Loffredo M., Dugal R., Hurwitz M., Kaplan I., Beard C.J., Renshaw A.A., Kantoff P.W. (2000). Biochemical Outcome Following External Beam Radiation Therapy with or Without Androgen Suppression Therapy for Clinically Localized Prostate Cancer. JAMA.

[21] Bolla M., Van Tienhoven G., Warde P., Dubois J.B., Mirimanoff R.-O., Storme G., Bernier J., Kuten A., Sternberg C., Billiet I. (2010). External irradiation with or without long-term androgen suppression for prostate cancer with high metastatic risk: 10-year results of an EORTC randomised study. Lancet Oncol..

[22] Bolla M., de Reijke T.M., Van Tienhoven G., Van den Bergh A.C.M., Oddens J., Poortmans P.M.P., Gez E., Kil P., Akdas A., Soete G. (2009). Duration of Androgen Suppression in the Treatment of Prostate Cancer. N. Engl. J. Med..

[23] Philip J.S., Matthew R.S. (2010). Adverse Effects of Androgen Deprivation Therapy: Defining the Problem and Promoting Health among Men with Prostate Cancer. J. Natl. Compr. Cancer Netw..

[24] Briganti A., Larcher A., Abdollah F., Capitanio U., Gallina A., Suardi N., Bianchi M., Sun M., Freschi M., Salonia A. (2012). Updated nomogram predicting lymph node invasion in patients with prostate cancer undergoing extended pelvic lymph node dissection: The essential importance of percentage of positive cores. Eur. Urol..

[25] Studer U.E., Collette L., Whelan P., Albrecht W., Casselman J., de Reijke T., Knönagel H., Loidl W., Isorna S., Sundaram S.K. (2008). Using PSA to guide timing of androgen deprivation in patients with T0-4 N0-2 M0 prostate cancer not suitable for local curative treatment (EORTC 30891). Eur. Urol..

[26] Spence W. (2018). Personalising Prostate Radiotherapy in the Era of Precision Medicine: A Review. J. Med. Imaging Radiat. Sci..

