# Supplementary material for: Should We Reconsider the Necessity of a Refinement of Prostate Cancer Risk Classification and Radiotherapy Treatment Strategy? Experiences from a Retrospective Analysis of Data from a Single Institution
Source: J Clin Med. 2020 Dec 30;10(1):110. doi: 10.3390/jcm10010110 (PMC7795563; doi:10.3390/jcm10010110)
Supplement: Supplementary file 1 [file jcm-10-00110-s001.pdf]

Supplementary Table 1. Properties of explanatory variables in the three risk groups respectively.

Explanatory variables	**Risk**	**Median (25%;75%)**	**Interquartile range**	**Minimum**	**Maximum**	**Skewness (Std. Error)**	**Kurtosis (Std. Error)**
**Delivered dose**	low	74.00 (74.00;87.00)	4.00	74.00	81.00	1.262 (0.403)	0.348 (0.788)
	intermediate	78.00 (78.00;78.00)	0.00	73.20	81.00	-0.517 (0.414)	3.435 (0.809)
	high	81.00 (74.40;81.00)	3.60	73.50	81.00	-1.002 (0.414)	0.179 (0.809)
**Plan accuracy (%)**	low	98.78 (97.87;99.25)	1.38	96.05	100.00	-0.784 (0.403)	0.105 (0.788)
	intermediate	98.57 (97.87;99.32)	1.44	94.24	99.99	-1.438 (0.414)	2.124 (0.809)
	high	97.95 (96.94;99.03)	2.12	92.12	99.50	-1.606 (0.414)	3.087 (0.809)
**ADT duration (months)**	low	6.0 (2.25;8.5)	6	0	60	3.239 (0.403)	12.711 (0.788)
	intermediate	22.0 (6;7.5)	30	6	60	0.685 (0.4149	-0.735 (0.809)
	high	36.0 (24.0;57.0)	33	6	120	1.376 (0.4149	4.125 (0.809)
**Time to reach PSA nadir (months)**	low	10.00 (5.00;26.00)	21	1	48	0.845 (0.403)	-0.284 (0.788)
	intermediate	13.00 (7.25;15.00)	8	1	35	0.724 (0.414)	1.358 (0.809)
	high	14.00 (8.00;18.00)	10	2	28	0.169 (0.412)	-0.779 (0.821)

Supplementary Table 2. Model summary of binary logistic regression (backward stepwise - likelihood ratio)

Model summary

Model	Chi-square	Sig.	Nagelkerke R square
1	7.492	0.278	0.146
2	0.000	0.995	0.146
3	-0.145	0.703	0.143
4	-0.300	0.584	0.138
5	-0.771	0.380	0.123
Step 1	Variables: (Constant), Delivered dose, Plan accuracy, Risk, ADT duration, Time to reach PSA nadir		
Step 2	Variables: (Constant), Delivered dose, Plan accuracy, Risk, ADT duration		
Step 3	Variables: (Constant), Plan accuracy, Risk, ADT duration		
Step 4	Variables: (Constant), Plan accuracy, Risk		
Step 5	Variables: (Constant), Risk		

Supplementary Table 3. Properties of explanatory variables in the four risk groups reclassified by the ISUP recommendations

Explanatory variables	Risk	Median	Interquartile range	Minimum	Maximum	Skewness (Std. Error)	Kurtosis (Std. Error)
**Delivered dose**	low	74.00 (74.00;87.00)	4.00	74.00	81.00	1.262 (0.403)	0.348 (0.788)
	intermediate 1	78.00 (78.00;78.00)	0.00	73.20	81.00	-0.592 (0.472)	3.227 (0.918)
	intermediate 2	78.00 (77.00;78.00)	1.00	76.00	81.00	0.872 (0.717)	2.589 (1.400)
	high	81.00 (74.40;81.00)	3.60	73.50	81.00	-1.002 (0.414)	0.179 (0.809)
**Plan accuracy (%)**	low	98.78 (97.87;99.25)	1.38	96.05	100.00	-0.784 (0.403)	0.105 (0.788)
	intermediate 1	98.64 (97.99;99.32)	1.33	95.34	99.87	-1.252 (0.472)	1.26 (0.918)
	intermediate 2	98.51 (97.35;99.44)	2.07	94.24	99.99	-1.370 (0.717)	2.601 (1.400)
	high	97.95 (96.94;99.03)	2.12	92.12	99.50	-1.606 (0.414)	3.087 (0.809)
**ADT duration (months)**	low	6.0 (2.25;8.5)	6	0	60	3.239 (0.403)	12.711 (0.788)
	intermediate 1	19.5 (6.0;36.0)	30	6	60	0.658 (0.472)	-0.596 (0.918)
	intermediate 2	30.0 (12.0;60.0)	48	6	60	-0.015 (0.717)	-2.220 (1.400)
	high	36.0 (24.0;57.0)	33	6	120	1.376 (0.4149	4.125 (0.809)
**Time to reach PSA nadir (months)**	low	10.0 (5.0;26.0)	21	1	48	0.845 (0.403)	-0.284 (0.788)
	intermediate 1	13.5 (5.0;26.0)	8	2	35	0.852 (0.472)	1.813 (0.918
	intermediate 2	13.0 (4.5;16.0)	12	1	24	0.034 (0.717)	-0.301 (1.400)
	high	14.0 (8.0;18.0)	10	2	28	0.169 (0.412)	-0.779 (0.821)
